# Effects of milling on texture and *in vitro* starch digestibility of oat rice

**DOI:** 10.1016/j.fochx.2023.100783

**Published:** 2023-07-06

**Authors:** Meng Shen, Kai Huang, Xiao Guan, Jian Xia, Zhu Sun, Zhiquan Yu, Yong Fang

**Affiliations:** a School of Health Science and Engineering, The University of Shanghai for Science and Technology, Shanghai 200093, PR China; b National Grain Industry (Urban Grain and Oil Security) Technology Innovation Center, Shanghai 200093, PR China; c Inner Mongolia Yangufang Ecological Agricultural Science and Technology (Group) Co., Ltd, Inner Mongolia, PR China; d College of Food Science and Engineering, Nanjing University of Finance and Economics, Nanjing 210003, PR China

**Keywords:** Milling, Oat rice, Texture, Sensory, *In vitro* starch digestibility

## Abstract

Compared with other oat products, consumers in China prefer oat rice and porridge made from naked oat. However, this oat product usually has poor sensory acceptance, which is directly related to the texture properties. This study aimed to use the milling method to improve the oat rice texture. The nutrient component, microstructure, pasting, and thermal properties of oat treated with different degrees of milling (0 s, 20 s, 40 s, 60 s, and 80 s) were researched. The results showed that milling would damage the bran layer of oat rice, increasing starch, β-glucan, total leached solids content, and the gelatinization enthalpy (ΔH). Meanwhile, oil, protein content, the pasting viscosity, and the pasting temperature were decreased. Milling made oat rice sticky and soft, and the bound water and non-flowing water migrated like flowing water. The cross-section of oat rice showed that milling damaged the surface of oat rice, which was beneficial to water entry and starch dissolution, and increased the viscosity of oat rice. When the milling time was 40 s and 60 s, the appearance, aroma, taste, texture, and overall acceptability of oat porridge were better. Moreover, rapid digestion fraction (k_1_) and slow digestion fraction (k_2_) are the lowest and have the effect of low blood glucose rise rate.

## Highlights

• Milling treatment reduced the oat porridge's pasting temperature and cooking time.
• Milling effectively improved the texture characteristics of oat rice.
• Moderate milling time was 40 s–60 s, nutrients, texture, and taste were better in this range.
• Milling had an impact on starch digestion, especially at 40 s and 60 s.

## Graphical abstract

**Figure:**
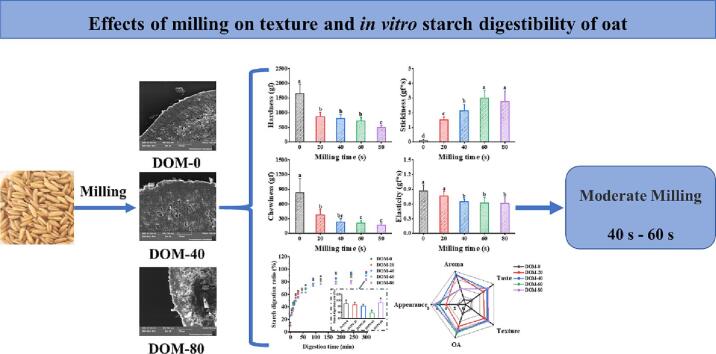

## Introduction

Oats (*Avena sativa*) contain large amounts of starch (about 60%), protein (13%–20%), oils (2%–12%), and β-glucan (2.0%–7.5%) (Zhang, Gao, Tong, & Li, 2018). There is evidence that oat plays a pivotal role in regulating obesity, anti-inflammatory, and antioxidation (Duan et al., 2022). Studies have shown that oat ethanol extract could effectively improve the status of serum, and liver lipids, and improve lipid metabolism disorders and hyperlipidemia (Huang et al., 2020). It has previously been observed that oat and their products have high nutritional benefits and are popular with consumers. However, naked oat has a hard taste, low viscoelasticity, and light oatmeal aroma. Many researchers aimed to study one type of oat rice with a soft texture and rich oat aroma conducive to consumer acceptance (Geng et al., 2020).

In Europe, North America, and other countries, oats are usually processed by baking bread or biscuits, while oats are usually used to prepare oat rice or noodles in China (Laaksonen, Ma, Pasanen, Zhou, Yang, & Linderborg, 2020). Naked oat poor cooking quality in terms of long cooking time, high hardness, less chewiness, and rough taste. Therefore, researchers use processing to improve its taste and texture characteristics and increase consumer acceptance. Compared with microwave or ultrasonic methods, milling reduced the seed layer and aleurone layer of oat rice but did not change the nutrient structure of oat rice (Walters, Udenigwe, & Tsopmo, 2018). Milling has the advantages of low cost and simple operation, which is conducive to processing oat products. Milling is a common grain processing method that ensures proper retention of grain nutrients and sensory properties. Many scholars hold the view that grain texture and nutritive properties could be improved by milling treatment (Geng et al., 2020). Milling appropriately removes the bran layer from the grain, which increases water absorption, decrease cooking time, and improves texture properties (Wang et al., 2016). The factors affecting the texture of oat are the cooking method and oat nutrition composition (Geng et al., 2020). Oat usually made into oatmeal or oat flour, oat flour was often mixed with wheat flour to make cookies, bread, and noodles. Oat rice and produces can be milled to change their whiteness and appearance. Different processing methods can improve the nutritional content and taste of oats, including baking, steaming, and boiling (Prates & Yu, 2017). Li et al. have shown that milling rice increased the whiteness and stickiness of the rice. The content of amylopectin (AP) and DP greater than 36 in the mill rice-leached materials were positively correlated with the viscosity (Li et al., 2021). The researchers observed that the rate and magnitude of the changes in the nutrient composition of brown rice caused by milling would help to optimize the DOM, meanwhile improving the character of brown rice cooking and consumption to better maintain nutrients (Longvah & Prasad, 2020). David et al. researched that milling reduced phenolic, flavonoid, and dietary fiber content, increased degrees of milling (DOM) and whiteness, and changed aroma composition. According to recent reports, compared with unpolished rice starches, milling speed and time can increase the swelling power (SP) and solubility of amylose (AM) (Xu et al., 2021). Researchers had shown that the milling process made brown rice lose and the network structure enlarged, while the contents of rapidly digestible starch (RDF) and slowly digestible starch (SDF) decreased and augmented, respectively (Li, Guan, & Li, 2021). Quinoa flour was added to wheat bread and noodles to reduce starch digestibility to some extent (Huang et al., 2022, Wang et al., 2021).

The purpose of this paper is to investigate the effects of milling treatment on the texture, gelatinization properties, and *in vitro* starch digestibility of oat rice. The changes in nutrient composition, water absorption index (WAI), SP, and total solids leaching during the cooking of oat rice with different DOM were also studied. The moisture distribution and molecular chain interaction of oat rice under DOM provided the basis for the WAI and SP of oat rice. The microstructure of the oat rice was observed by scanning electron microscope (SEM). Determination of functional group changes of oat rice with milling treatment by Fourier transform infrared (FTIR) spectroscopy. Differential scanning calorimeter (DSC) and Rapid Visco-Analyzer (RVA) techniques were used to study the pasting and thermal properties of oat during different DOM. Pearson correlation analysis was used to analyze the relationship between the correlation index and texture index in the cooking process. The appearance, aroma, taste, texture, and overall acceptability were evaluated through sensory evaluation experiments to determine the optimum milling time. Combined with the conclusion of sensory evaluation and *in vitro* digestion of starch, aiming to develop nutritionally fortified oat rice with both slower starch digestibility and desirable textural attributes. This study provided the theoretical basis for oat in the food industry.

## Materials and methods

### Materials

Whole-grain oat was purchased from Yangufang Ecological Agricultural Science and Technology Co., Ltd. (Inner Mongolia, China). Total starch, AM/AP, and mixed-linkage beta-glucan kits were purchased from Megazyme International Ireland Ltd. (Bray Co., Wicklow, Ireland). The BCA protein assay kit was purchased from Biyuntian (Shanghai, China). Other chemicals were analytical grades.

### DOM

The oat was milled by a grain polisher (NA-JCB machine, Zhengzhou Zhonggu Machinery Equipment Co., Ltd., Henan province, China) for 0 s, 20 s, 40 s, 60 s, and 80 s, respectively. These samples are named DOM-0, DOM-20, DOM-40, DOM-60, and DOM-80. The oat rice was ground into powder through a 100 mesh sieve and stored at 4 ℃.

$$\mathrm{DOM}\;\left(\%\right)\;=\left(1-\frac{\mathrm{weight}\;of\;milled\;oat\;rice\;\left(g\right)}{\mathrm{weight}\;of\;oat\;rice\;\left(g\right)}\right)\times 100\%$$

### Oat rice whiteness and composition analysis

Oat rice samples were rinsed three times with distilled water in and 45 ℃ oven for 24 h drying. Place the oat rice in a petri dish about 3 cm high. A tristimulus colorimeter (Konica Minolta Holdings, Inc., Tokyo, Japan) was used to obtain lightness (L), redness (a*), and yellowness (b*) (Wu et al., 2023, Ziegler et al., 2018). The calculation formula is as follows:

$$\mathrm{Whiteness}=100-\sqrt{{\left(100-L\right)}^{2}+a^{2}+b^{2}}$$

The protein and fat contents were evaluated by the Kjeldahl method (Lynch & Barbano, 1999) and Soxhlet extraction (Saim et al., 1997), respectively. Starch and β-glucan were evaluated by kit (Huang et al., 2022, McCleary and Codd, 1991).

### SEM and FTIR analysis

The transverse section of oat rice with different DOM was evaluated using an SEM (TM-1000, Hitachi, Japan) (Wang, Wu, Zhang, Kan, & Zheng, 2022). After spraying gold, the accelerated voltage of the sample was 30 kV, and the sample was amplified 500× for observation.

According to the previous research method, the sample and KBr were completely ground and pressed, and FTIR infrared spectrometer (Nicolet IS10, Thermo Fisher Scientific, Waltham, MA, USA) was used to scan the spectrum from 400 cm^−1^ to 4000 cm^−1^ (Su et al., 2020).

### Pasting and thermal properties analysis

Accurately weigh a 3.0 g (dry basis) sample in RVA (RVA-TecMaster, Perten Instruments, NSW, Australia) tank, add distilled water, and quantify to 28.0 g. The pasting characteristics of oat rice were determined according to the research method proposed by Qian et al (H. Y. Li, M. H. Xu, Z. J. Chen, J. Li, Y. Y. Wen, Y. L. Liu, et al., 2021; Qian, Sun, Zhu, Zhang, Tian, & Wang, 2020).

3 mg (dry basis) samples were accurately weighed, pressed with 6 µL of distilled water, and at room temperature stand for 12 h. The change of ΔH, onset temperature (To), peak temperature (Tp), and conclusion temperature (Tc) were measured according to the method of Yang et al (Yang, Chi, Liu, Zhang, Zhang, & Wang, 2019; Y. Zhang, Li, Huang, Li, Cao, Xie, et al., 2023).

### SP and WAI measurement

The Li method has been slightly modified (F. Li, Guan, & Li, 2021). The oat rice (Wi) and distilled water were placed in a centrifuge tube at 1:15 (w/v) and boiled water bath for 10 min. Then the sample was cooled to room temperature and centrifuged at 7000 × g for 10 min, precipitation (W*r*) and dry supernatant (Ws) were weighed.

$$\text{SP (g/g) =}\;\frac{{\text{W}}_{\text{r}}}{{\text{W}}_{\text{i}}\text{-}{\text{W}}_{\text{s}}}$$

$$\text{WAI (g/g) =}\;\frac{{\text{W}}_{\text{r}}}{{\text{W}}_{\text{i}}}$$

### Determination of moisture distribution by Low-Field nuclear magnetic resonance (LF–NMR)

The boiled oat rice was put into a nuclear magnetic tube, and T_2_ was determined by the GPMG sequence. Each sample was repeated twice. CPMG sequence parameters are set as spectrometer frequency (SF) 23 MHz, offset frequency (O1) 312.3755 kHz, 90° pulse time P90 14 µs, 180° pulse time P180 28 µs, number of sampling points (TD) 40020, repeated sampling waiting time (TR) 4500 ms, scanning number (NS) 64, echo time (TE) 200 µs, echo number Echo Count 1000, analog gain (RG) 20.0 dB, digital gain (DRG) 3 (Bin Karim et al., 2021, Gallego-Lobillo et al., 2021, Zhang et al., 2022).

### Total solids leached from cooked oat rice and composition analysis

The previous research methods have been slightly modified (H. Y. Li et al., 2021). Add distilled water (95 ℃) to cooked oat rice in a ratio of 1:5 (w/v). Strain through a 250 μm sieve and rinse twice repeatedly with 50 mL distilled water (95 °C). The filtrate was freeze-dried and weighed (Li, Guan, & Li, 2021). Component analysis was determined following Method 2.3.

### Texture profile analysis (TPA)

Placed 2.5 g of cleaned oat rice in a beaker, added distilled water at 1:6 (W/V), and cooked in an induction cooker (1600 W) for 60 min. Take the middle of the beaker and mix the oat rice for determination. The TPA was measured by a P/36R cylindrical probe. Pre-test speed 10.0 mm/s, Test speed 1.0 mm/s, Post-test speed 1.0 mm/s, Target mode Strain, Strain 75%, Time 5.0 s, Trigger force 5.0 g. The hardness, stickiness, chewiness, and elasticity were selected as the detection indexes of the texture of oat rice (Wang, Cheng, Li, Li, Hong, & Gu, 2022). Each measurement has 10 replicates.

### Sensory evaluation

Oat porridge was cooked in the same way as 2.9 TPA. 15 panelists (8 females, and 7 males, aged 23–30) had background and knowledge in food science or had experience in sensory analysis and were selected to participate in this study. Rank products based on appearance, taste, texture, aroma, and overall acceptability on a scale of 1 (strongly dislike) to 9 (strongly like). Each reviewer provided mineral water for mouthwash.

### *In vitro* digestion of cooked oat porridge flour

50 mg oat porridge powder and 2 mL distilled water were placed in a centrifuge tube and bathed at 37 ℃ for 5 min, and 8 mL of trypsin and starch glycoside enzyme buffer were added, then shaken in a 37 ℃ water bath shaker. At 0 min, 5 min, 10 min, 15 min, 20 min, 30 min, 45 min, 60 min, 90 min, 120 min, 180 min, 240 min, and 300 min, take 100 μL and immediately mixed with 900 μL anhydrous ethanol and centrifuge the supernatant. Glucose oxidase/peroxidase reagent (GOPOD) was used for determination (Huang et al., 2022, Li et al., 2021).

### Logarithm of slope plot (LOS) and combination of parallel and sequential digestion kinetics (CPS) analysis

The logarithm formula of the logarithm of slope (LOS) plot was used to fit the starch digestion map of milled and defatted oat rice.

$$\ln\frac{dC_{t}}{\mathrm{dt}}=\ln\left(C_{\infty }-C_{0}\right)-kt$$

C_t_ is the starch fraction digested at time t (min), while C_0_ is the starch fraction digested at t = 0. C_∞_ is the estimated maximum starch digestibility, and k is the digestion rate constant.

The LOS curve can determine the number of starch components with different digestible rate constants. CPS model can distinguish various digestive patterns in the starch digestive system (Butterworth, Bajka, Edwards, Warren, & Ellis, 2022).

$$C\left(t\right)=C_{0}+\left(C_{\infty 1}\right)\times \left(1-e^{-k_{1}t}\right)+If\left(t⩾t_{2start},\left(\left(C_{\infty 2}\right)\times \left(1-e^{-k_{2}\left(t-t_{2start}\right)}\right)\right)\right),0$$

In the above equation, k_1_ and k_2_ respectively represent the digestion rate coefficients, and C_∞1_ and C_∞2_ respectively represent the maximum digestibility of the two starch components. t_2start_ represents the point in time at which partial digestion of the second starch begins. The digestive patterns of rapid and slow starch components could be determined by t_2start_. When t_2start_ = 0, t_2start_ ≥ t_1_ (completion time of rapid digestion), and 0 < t_2start_ < t_1_, represent parallel mode, sequential mode, and combination mode respectively (Butterworth et al., 2022). CPS model parameters were quantified by the nonlinear least square method.

### Statistical analyses

All the results were expressed as mean value ± SD (standard deviation). A descriptive analysis of ANOVA, correlation analysis, and heat map clustering analysis was performed using SPSS Statistics software version 19.0 (SPSS, Chicago, IL) and Origin 2021 (OriginLab, Northampton, MA, USA).

## Results and discussion

### Effect of DOM on oat rice whiteness, compositions, SP, and WAI

Table S1 shows that there has been an increase in the number of DOM, whiteness, β-glucan, and starch level of oat rice with the milling time increased. However, the content of protein and oil were reduced. The sub-aleurone layer and endosperm of oats contain a lot of starch and protein. Increasing DOM removed pigment, protein, fiber, and oil from oat bran, therefore, the cooking time was reduced. Meanwhile, oat rice or oat porridge became soft, thick, and waxy. Li and Xu et al. researched that whiteness and stickiness could increase by milling treatment, and confirmed that the increase of starch pasting degree and the reduction of leached AM would produce this condition (Li et al., 2021).

Milling destroyed the layer, and increased the diffusion of water and the pasting of starch during cooking (Zhu et al., 2020), while the fiber and protein have a strong tensile strength in the starchy endosperm (Li, Guan, & Li, 2021). Meanwhile, milling increased the starch content and damaged the oat rice epidermis, which conducted water entry and starch gelatinization. Therefore, milling increased the SP and WAI of oat rice (Table S2). Accompanying the change in oat rice components, the morphology and structure of oat rice were damaged by milling treatment (Peng et al., 2020).

### SEM observation and FTIR analysis

**Fig. 1:**
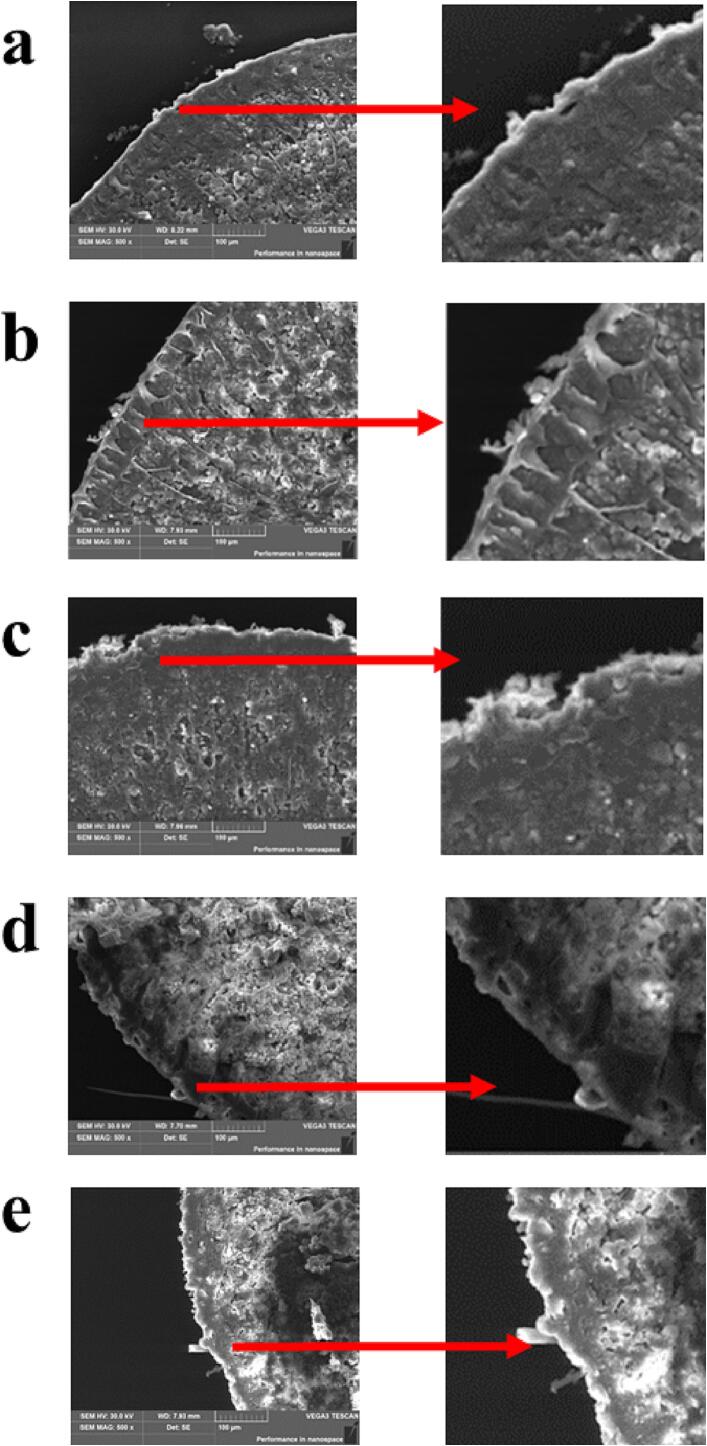
SEM of the transverse cross-section of oat rice with different DOMs (a-e: 0 s, 20 s, 40 s, 60 s, and 80 s).

Fig. 1 shows the cross-section of oat rice. The seed coat of untreated oat rice is intact, and the particles are still tightly aggregated. Milling (20 s-80 s) destroyed the oat seed coat structure and removed the bran layer (Liu, Zhao, Zhang, Wang, & Liu, 2021). As the milling time increases, the surface of the milled oats is more severely damaged, and the aleurone layer is removed to a certain extent (Meziani et al., 2021). The processing properties and texture characteristics of the oat rice were consistent with the results of the whiteness, composition, WAI, and SP of milled oat rice to different degrees. According to SEM, when the milling time was 0 s, 20 s, and 40 s, the seed coat of the oat rice surface was completely removed, which would increase the starch dissolution of the aleurone layer of oat rice. When the milling time was 60 s and 80 s, the aleurone layer was partially removed. During cooking, starch gelatinization, and starch dissolution rate of oat rice were improved, increasing the WAI, SP, and viscosity of oat rice were increased, improving the viscosity of oat rice, the hardness of oat rice was reduced (Li et al., 2021).

**Fig. 2:**
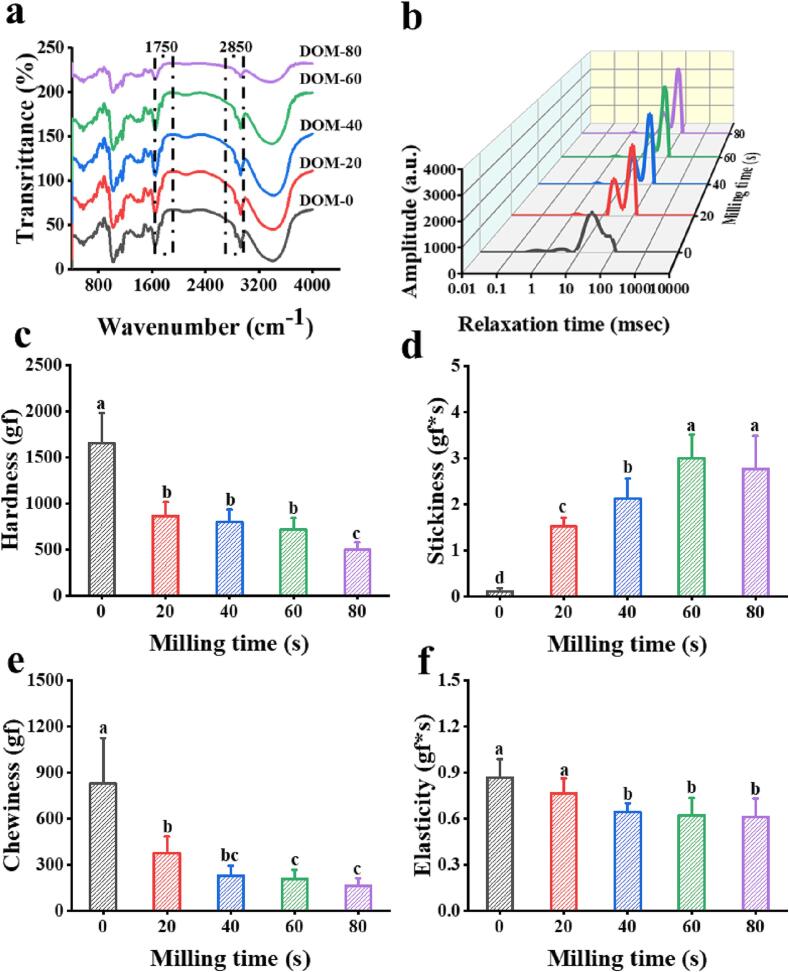
FITR (a), distribution of transverse relaxation time (T_2_) (b), hardness (c), stickiness (d), chewiness (e), and elasticity (f) of oat rice with different DOMs.

Fig. 2 (a) shows FTIR spectra of milling oat rice. Asymmetric tensile vibrations of fatty acids –CH_3_ and –CH_2_ at absorption peaks near 2850 cm^−1^. The vibration of C=O is around 1750 cm^−1^, and the peak of C=O of fatty acids is around 1700 cm^−1^ (Zheng et al., 2018). The formation of hydrogen bonds between C=O and OH of AM causes a peak blue shift (Li et al., 2021). The characteristic absorption peaks at 1735 cm^−1^ and 2850 cm^−1^ gradually weakened as the milling treatment increased. Because of milling made the oil content in oat rice was effectively reduced, and the starch-lipid combination was reduced, which would affect the texture and gelatinization characteristics of oat rice (Li, Obadi, Shi, Xu, & Shi, 2021). Fig. 2 (a) shows that there was no chemical change in the milling of oat rice, and no new characteristic absorption peaks appeared or disappeared. The results also indicate that there was no chemical reaction between starch and oil, but a stable complex was formed through hydrophobic complexation.

### LF-NMR analysis of cooked oat rice with different DOMs

Fig. 2 (b) is the inversion spectrum of the transverse relaxation time T_2_ of different DOM oats during the cooking process. The inverted NMR T_2_ spectrum of oat has 3 peaks, representing 3 different water states of oat. And the range of transverse relaxation time T_2_ is T_21_ (0.1–1 ms), T_22_ (1–10 ms), and T_23_ (10–1000 ms) (Bin Karim, Ordoubadi, Wang, Gomez, & Vehring, 2021). The transverse relaxation time T_2_ is the time required for the spin nucleus of the H proton in the external magnetic field to reach the transverse thermal equilibrium of the system after receiving the RF pulse stimulation (Gallego-Lobillo, Doyaguez, Jimeno, Villamiel, & Hernandez-Hernandez, 2021). Studies have shown that T_21_ represented bound water, T_23_ represented free water, and T_22_ was adsorbed water between bound water and free water, which was not easy to flow (Zhang, Sun, Gao, & Luo, 2022). T_2_ is proportional to the mobility of the water molecules. The shorter T_2_ is, the less liquid the water is, and the ability to bond with water increases. The peak areas of T_21_, T_22_, and T_23_ (M_21_, M_22_, and M_23_) can represent the signal amplitudes of water in various states. The proportions of M_21_, M_22_, and M_23_ to the total area are S_21_, S_22_, and S_23_, respectively, which can indirectly reflect the water in different states and moisture content (Iskandar et al., 2021).

It can be seen from Fig. 2 (b) and Table S3 that the bound water content decreases with the milling time increases, meanwhile, the peak position in the corresponding T_2_ relaxation spectrum pushes to a longer relaxation time. This result indicates that the strength of organic material binding to water generally tends to weaken with the entry of water molecules (Iskandar, et al., 2021).

### Total solids leached from cooked oat rice and composition analysis

What is interesting in this Table S4 is the leached solids content of milled oat rice increases with increasing DOM. This indicates that the fiber of the oat bran was destroyed, and the aleurone layer was exposed by milling. The leached solids of the oat rice were easier to dissolve, which was consistent with a previous report (Li, Guan, & Li, 2021). The composition analysis of the leached solids showed that the starch content and AP content were significantly increased by milling treatment. Compared with unmilled oat rice (DOM-0), the AP content of DOM-80 was more than 6 times, which was consistent with previous research results (Li et al., 2021). The milling time was 0 s and 20 s, the protein, and β-glucan increased, whereas, when the milling time was 40 s, 60 s, and 80 s, the protein and β-glucan decreased by 33.72% and 63.39%, respectively. The oat bran layer contains a lot of protein and β-glucan, and milling treatment reduces the bran layer (Zhu et al., 2020). Starch content was the highest in the extract, and the AP content and stickiness were increased by milling treatment (Li et al., 2021).

### TPA

Fig. 2 (c–f) shows the texture properties of cooked oat rice with milling treatment. Hardness, stickiness, chewiness, and elasticity were selected as the main contributing factors for comparison (Wang et al., 2022). Fig. 2 (c–f) is the rapid decrease in hardness, chewiness, and elasticity of the cooked oat rice, however, the stickiness is just the opposite, when milling time increases. The hardness of the oat rice was reduced. It is because milling increased the starch content of oat rice and reduced the fiber, protein, and oil content of oat rice.

The starch content in the leached solids was higher, and the proportion of AP increased, which in turn increased the stickiness of oat rice. When the DOM increased, the oil content of the oat rice decreased, and the number of starch-lipid complexes formed during the cooking process decreased. And the newly formed complexes may be more concentrated at the endosperm-bran interface, which is rich in lipids and proteins, and the hardness and elasticity of cooked oat rice were reduced (Li, Guan, & Li, 2021). Undercooking conditions, the oat starch gelatinized and amylose–lipid complexes were formed, then the starch regrows when it is cooled and dried. The degree of starch regeneration and the degree of complexity with lipids decreased with the DOM increased, which may reduce the hardness and elasticity of oat rice (Wang et al., 2022).

### Pasting properties

The RVA paste properties of oat rice with milling treatment are in Table 1. The pasting properties of oat flour are primarily determined by the oat starch but are also affected by other components such as proteins, lipids, and β-glucans (Qian et al., 2020). Compared with DOM-0, milling increased the peak viscosity of oat rice (P > 0.05). When the milling time was 60 s of milled oat rice, the trough viscosity, final viscosity, peak time, and pasting temperature reached the lowest value (1649.00 cP, 3524.50 cP, 6.07 min, and 60.08 ℃). Previous research suggested that oats contain high lipids amounts, which can compound with amylose, slowing down or even hindering the expansion of starch particles. This phenomenon resulted in reduced starch solubility, delayed pasting, and limited gel creation, as well as higher pasting temperature (Gomez-Aldapa et al., 2020). The starch content of the oat was enhanced and the lipid level was reduced by milling, the AM/AP ratio was also changed, so the pasting temperature of oat flour showed a downward trend. This is similar to the previous results in Table S1. When the temperature began to drop, long-chain starch molecules began to cross-link with each other to form a network structure, and AM was easier to cross-link with each other due to fewer branches (Cao et al., 2022). Therefore, milling would reduce the final viscosity of oat rice. When the peak viscosity and breakdown viscosity of the grain was high, the setback viscosity was low, and it will be more sticky and soft after cooking.

**Table 1:** Pasting and thermal properties of oat rice with milling.

Milling time (s)	0	20	40	60	80
**Pasting properties**					
Peak viscosity (cP)	2617.50 ± 24.75^a^	2668.50 ± 9.19^a^	2653.00 ± 76.37^a^	2662.00 ± 15.56^a^	2738.00 ± 110.31^a^
Trough viscosity (cP)	2020.50 ± 47.38^a^	1746.00 ± 4.24^bc^	1649.50 ± 40.31^c^	1649.00 ± 7.07^c^	1767.50 ± 71.42^b^
Breakdown viscosity (cP)	597.00 ± 22.63^c^	922.50 ± 4.95^b^	1003.50 ± 36.06^a^	1013.00 ± 8.49^a^	970.50 ± 38.89^ab^
Final viscosity (cP)	4791.50 ± 20.51^a^	4353.50 ± 44.55^b^	3657.00 ± 32.53^c^	3524.50 ± 44.55^d^	3559.50 ± 81.32^cd^
Setback viscosity (cP)	2771.00 ± 26.87^a^	2607.50 ± 48.79^b^	2007.50 ± 7.78^c^	1875.50 ± 51.62^d^	1792.00 ± 9.90^d^
Peak time (min)	6.57 ± 0.05^a^	6.30 ± 0.04^b^	6.30 ± 0.04^b^	6.07 ± 0.00^c^	6.30 ± 0.04^b^
Pasting temperature (°C)	63.68 ± 0.04^a^	61.00 ± 0.07^b^	60.15 ± 0.07^b^	60.08 ± 0.04^c^	60.20 ± 0.00^b^
**Thermal Properties**					
T_o_ (°C)	57.41 ± 1.01^a^	57.24 ± 0.83^a^	56.97 ± 1.04^a^	56.83 ± 0.80^a^	56.60 ± 1.47^a^
T_p_ (°C)	63.90 ± 1.03^a^	63.28 ± 0.32^a^	63.04 ± 0.12^a^	62.62 ± 0.52^a^	62.58 ± 0.07^a^
T_c_ (°C)	72.82 ± 1.22^a^	72.32 ± 1.31^a^	71.99 ± 0.25^a^	71.30 ± 1.22^a^	71.64 ± 0.58^a^
ΔH (J/g)	5.65 ± 0.19^a^	5.92 ± 0.21^a^	5.58 ± 0.49^a^	5.56 ± 0.26^a^	5.87 ± 0.44^a^

Note: T_o_: temperature onset, T_p_: temperature peak, T_c_: temperature conclusion, and ΔH: enthalpy. The values of different letters in each row indicated a significant difference between milling treatment with oat rice (p < 0.05).

### Thermal properties

What can be seen in this Table 1, the T_o_ and T_p_ temperature of oat rice decreased with the increase in milling time, and the T_c_ temperature was the lowest when the milling time was 60 s. Gelatinization ΔH rose to a high point and peaked in milling time is 20 s. Studies have shown that the increase in AM content leads to the decrease of ΔH; A larger proportion of AP leads to more double helices in the crystallization domain of particles, increasing ΔH (Bai, Zhou, Zhu, & Li, 2021). The molecular structure of AP (unit chain length and branching degree), AM/AP ratio, and particle structure (ratio of crystallization to amorphous) all affect T_o_ (Yang et al., 2019). When milling time was 20 s and 80 s, AM content was higher in oat porridge. In the heating process, the AP regrouped and formed a more orderly and stable double helix, which improved the stability. So the crystallinity of starch crystal melting energy needed for the double helix structure of the starch chain increased ed, and more energy was required to destroy the starch granule structure (Su, et al., 2020). The decrease in pasting temperature is due to the binding between AM-AM and AM-lipid (Acquisgrana et al., 2020). It is consistent with the previous conclusions.

### Sensory evaluation

**Fig. 3:**
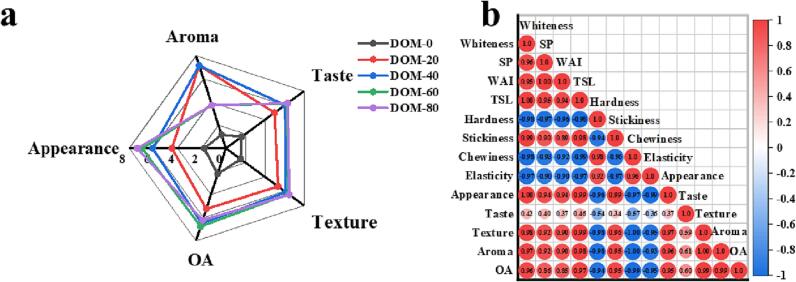
Sensory evaluation of cooked oat rice with different DOMs (a); Correlation analysis between total solids leached, SP, WAI, hardness, stickiness, chewiness, elasticity, and sensory evaluation of oat rice with different DOM (b) (OA: overall acceptability; TSL: total solids leached).

Sensory evaluation of oat rice is necessary to determine consumer acceptance of the practical applications of the DOM. The results of the sensory assessment of oat rice are shown in Fig. 3 (a). Milling time increased the appearance of oat porridge, with aroma peaking at 20 s and 40 s. And taste, texture, and overall acceptability reached maximum values at 40 s, 60 s, and 80 s without significant differences. In conclusion, although milling reduces some nutrients of oat rice, it can greatly improve the appearance, taste, and texture of oat porridge. Therefore, it is suggested that the milling time of oat rice should be 40 s and 60 s.

### Correlation analysis

Correlation analysis was used to measure whether there was synergy or antagonism between different indicators. In this study, Origin software was used to analyze the correlation of 7 indicators after cooking oat rice with different milling times, and the Pearson correlation coefficient method was used (this method is mainly used to measure the degree of correlation between two variables), the statistical results are shown in Fig. 3 (b). The whiteness of oat rice was significantly positively correlated with the appearance and overall acceptability of oat porridge. Hardness, chewiness, and elasticity were negatively correlated with appearance, texture, aroma, and overall acceptability, while the opposite of viscosity. The total solid leached, SP, and WAI indicated a significant negative correlation with hardness, chewability, and elasticity, while the total solid leached, SP, and WAI indicated a significant positive correlation with viscosity, appearance, texture, aroma, and overall acceptability. The results indicated that the milling treatment enhanced the overall acceptability of oat porridge and was beneficial to the acceptability of oat porridge.

### *In vitro* starch digestion of milling oat porridge

**Fig. 4:**
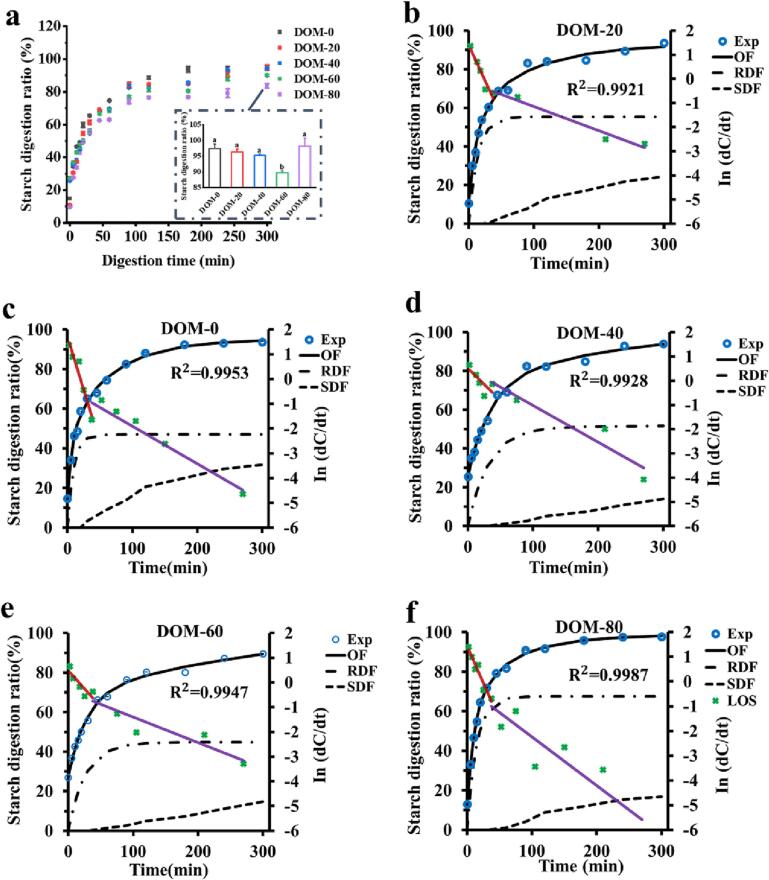
Starch digestion for oat porridge with different DOMs (a) and their corresponding LOS/CPS kinetics model fittings (b-f). Exp is experimental data. OF is the overall fit curve.

Fig. 4 (a) shows the digestion curves of oat porridge with different DOMs. Each digestion curve showed exponential growth and 0–30 min was the rapidly digestible starch fraction (RDF). Maximal digestibility was slowly reached after 120 min. The digestion of oat porridge accorded with first-order kinetics models. The maximum starch digestibility of all samples was higher than 90%. The higher digestibility results from the destruction of the starch crystalline structure during cooking. Milling treatment reduced the starch digestion rate of oat porridge to some extent. It has been reported that milling will increase the digestion rate of brown rice due to the destruction of the bran layer, increasing the WAI of rice, the degree of starch gelatinization, the distance between starch molecules, etc (Li, Guan, & Li, 2021). The contrary results of this study may be because oats contain higher oil, and the oil distribution of oat could be changed at the same time when the oil was reduced by milling (Zhen et al., 2022). During cooking, starch, and oil form an AM lipid complex, which will reduce the starch digestion rate (Sun et al., 2021).

First-order kinetic models are often used to study starch digestion in different food systems. LOS and CPS kinetic models are used for fitting. For systems containing multiple starch-digestible components, a CPS digestion kinetic model is commonly used. The sequential digestion pattern is generally suitable for food systems with RDF outside and slowly digestible starch fraction (SDF) inside (Zhen et al., 2022). SDF starts to digest only after RDF digestion is complete (Wang, Lao, Bao, Guan, & Li, 2021). Food systems in which the two starch components are digested simultaneously but at different rate constants use parallel digestion patterns (Huang, Huang, Guan, Zhang, & Zhang, 2022). The curve of Fig. 4 (b-f) shows that the digestion of oat porridge is divided into two discontinuous components, which indicates that there are two starch components with different digestion rate constants in the digestive system. By analyzing digestibility, the oat porridge digestive system can be divided into RDF and SDF. After fitting, the R^2^ of all samples was greater than 0.99, indicating that the fitting results were credible. According to the CPS model fit, t_2start_ was within 0 min of the end of digestion in the first digestive phase. This indicated that the digestion of samples was carried out by a CPS digestion. As Table 2 shows that, milling treatment had a significant effect on the RDF (k_1_) rate and SDF (k_2_) rate constant, especially for DOM-0 k_1_, which was significantly 3 times higher than that of DOM-40. The maximum starch digestibility of RDF (C_∞1_) increased and then decreased, while that of SDF (C_∞2_) was just the opposite. The content of resistant starch (RS) in oat porridge significantly increased with milling treatment. Therefore, the initial digestion time of SDF (t_2start_) increased first and then decreased. Due to the increased starch and reduced lipid content in the milling of oat rice, amylose–lipid complexes will be formed during the cooking process, which will affect the digestibility of starch (Qin et al., 2019, Zhen et al., 2022). Researchers have shown that the heating method can form starch complex with oil, reduce the digestibility of starch, and increase the digestibility of starch with the decrease of oil content (Tang, Wang, Cheng, Wu, & Ouyang, 2019). Interestingly, on the contrary, the results of this study showed that the oil content of oats decreased, the starch content increased, and the starch digestibility showed a decreasing trend. This result may be because β-glucan is the main influencing factor in the process of starch digestion. The natural network structure of β-glucan can encapsulate protein and starch, and it has a certain resistance to amylase, which reduces the accessibility of the enzyme and thus reduces the digestion of starch (Zhang, Luo, & Zhang, 2017).

**Table 2:** Fitting parameters of CPS dynamic model.

Milling time (s)	0	20	40	60	80
RS (%)	11.29 ± 0.82^d^	15.50 ± 0.58^c^	17.49 ± 0.18^b^	18.77 ± 1.23^b^	23.55 ± 0.52^a^
k_1_ × 10^-2^	10.35 ± 0.26^a^	7.14 ± 0.13^c^	2.95 ± 0.09^e^	4.09 ± 0.28^d^	7.73 ± 0.00^b^
C_∞1_ (%)	48.25 ± 1.19^c^	56.38 ± 0.96^a^	52.55 ± 0.90^b^	43.33 ± 1.24^d^	48.85 ± 0.35^c^
t_2start_ (min)	24.56 ± 1.95^b^	28.63 ± 0.17^a^	30.09 ± 0.15^a^	29.92 ± 0.10^a^	25.01 ± 0.55^b^
k_2_ × 10^-2^	1.37 ± 0.06^a^	0.97 ± 0.11^b^	0.47 ± 0.12^c^	0.42 ± 0.16^c^	1.15 ± 0.05^b^
C_∞2_ (%)	33.30 ± 0.13^a^	27.92 ± 0.85^b^	18.87 ± 1.18^d^	26.94 ± 0.48^b^	24.11 ± 1.14^c^

Note: RS: resistant starch. The values of different letters in each row indicated a significant difference between milling treatment with oat rice (p < 0.05).

## Conclusion

This study set out to impact DOM on nutrient ingredients, pasting, thermal, texture properties, and starch digestion. The milling process increased stickiness, β-glucan, and the whiteness of oat rice, but decreased hardness, chewiness, elasticity, protein, and lipids content. Milling also increased the total solid leached and leached starch content but decreased the leached protein content. It is shown that milling damaged the oat bran layer, reduced AM and starch-lipid interaction, and reduced pasting temperature and gelatinization viscosity. DOM increased the level of starch and β-glucan leaching while reducing the bran layer at the oat, resulting in increased viscosity during rice milling. Through sensory evaluation experiments, it was finally determined that 40 s and 60 s were the most suitable milling periods, with higher relative scores for appearance, taste, aroma, texture, and overall acceptability. Pearson analysis found that the WAI, SP, and total solid leached of oat rice were related to the texture properties. According to the analysis of starch digestion in oat porridge with milling treatment, when the milling time is 40 s–60 s, k_1_ and k_2_ are the lowest, while t_2start_ is the highest, indicating that the digestion rates of RDF and SDF are lower at this time, which is suitable for patients with hyperglycemia. Consequently, proper milling can improve the texture properties and starch digestibility of oat. Combined with the conclusion of sensory evaluation and *in vitro* digestion of starch. The present study designed oat rice with higher nutrient intensity, better texture, and slower starch digestibility.

## Declaration of Competing Interest

The authors declare that they have no known competing financial interests or personal relationships that could have appeared to influence the work reported in this paper.

## Data Availability

The data that has been used is confidential.
